# From reverse innovation to global innovation in animal health: A review

**DOI:** 10.1016/j.heliyon.2021.e08044

**Published:** 2021-09-21

**Authors:** Lisa Crump, Yahya Maidane, Stephanie Mauti, Rea Tschopp, Seid Mohammed Ali, Rahma Abtidon, Hervé Bourhy, Zakaria Keita, Seydou Doumbia, Abdallah Traore, Bassirou Bonfoh, Mathilde Tetchi, Issaka Tiembré, Vessaly Kallo, Vega Paithankar, Jakob Zinsstag

**Affiliations:** a Swiss Tropical and Public Health Institute, PO Box, 4002, Basel, Switzerland; b University of Basel, Petersplatz 1, 4003, Basel, Switzerland; c Jigjiga University, Jigjiga, Ethiopia; d Institut Pasteur, 25-28 Rue du Dr Roux, 75015, Paris, France; e Université des Sciences, des Techniques et des Technologies de Bamako, BP, 1805, Bamako, Mali; f Laboratoire Central Vétérinaire, Sotuba, Bamako, Mali; g Centre Suisse de Recherches Scientifiques en Côte d'Ivoire, 01 BP, 1303, Abidjan, Cote d'Ivoire; h Institut National d'Hygiène Publique, 23 BP, 3838, Abidjan, Cote d'Ivoire; i Ministère de Resources Animales et Halieutiques, Abidjan, Cote d'Ivoire; j Health Information Traceability Stiftung, Gotthardstrasse 26, Zug, Switzerland; k Armauer Hansen Research Institute, PO Box 1005, Addis Ababa, Ethiopia

**Keywords:** Reverse innovation, Digital innovation, Social innovation, Contact sensor, Rapid diagnostic test, Integrated surveillance-response system

## Abstract

Reverse innovation refers to learning from or diffusion of innovations developed in low income settings and further translated to industrialized countries. There is lack of consensus regarding terminology, but the idea that innovations in low-income countries are promising for adoption in high-income contexts is not new. However, in healthcare literature globally, the vast majority of publications referring to ‘disruptive innovation’ were published in the last ten years. To assess the potential of innovative developments and technologies for improving animal health, we initiated a literature review in 2020. We used a combined approach, incorporating targeted searching in PubMed using a key word algorithm with a snowball technique, to identify 120 relevant publications and extract data for qualitative coding. Heterogeneity of articles precluded meta-analysis, quality scoring and risk of bias analysis. We can distinguish technical innovations like new digital devices, diagnostic tests and procedures, and social innovations of intersectoral cooperation. We profile two case studies to describe potential global innovations: an integrated surveillance and response system in Somali Regional State, Ethiopia and a blockchain secured One Health intervention to optimally provide post-exposure prophylaxis for rabies exposed people in West Africa. Innovation follows no borders and can also occur in low-income settings, under constraints of cost, lack of services and infrastructure. Lower administrative and legal barriers may contribute to produce innovations that would not be possible under conditions of high density of regulation. We recommend using the term global innovation, which highlights those emanating from international partnership to solve problems of global implications.

## 1 Introduction

The term ‘reverse innovation’ (RI) originated in industry built on the idea of ‘disruptive innovation’ (Bower and Christensen, 1995) and was introduced in management literature 15 years later (Immelt et al., 2009) and further popularized (Govindarajan and Trimble, 2012). An informative recent review of multidisciplinary literature analysed definitions of RI, proposing a conceptual framework which highlights managerial implications (Hadengue et al., 2017). Notably, that review did not include the PubMed database, and significant differences in meaning and use exist relative to health policy circles, where the term has gained traction. In healthcare, RI refers to learning from or diffusion of innovations developed in low income settings and further translated to industrialized countries. This more complex, fragmented process (Harris et al., 2016b), is conflated with other terms like ‘frugal’ or ‘trickle-up’ innovation, ‘leapfrog technology’ or ‘innovation blowback’, and the literature poses varied definitions (Skopec et al., 2019). Frugal innovation describes the concept of altering existing systems (disrupting) by doing better with less (Bolton et al., 2019). Leapfrogging refers to using informal knowledge (based on experience, intuition and interaction) to propel development (Mormina, 2019), while innovation blowback highlights unexpected consequences of development in emerging markets (Brown and Hagel, 2005). More recently, the term reverse innovation broadens to include innovations that originate in high-income (HI) countries but are tested and scaled up in low-and middle-income (LMIC) countries and subsequently re-enter HI markets (Snowdon et al., 2015; von Zedtwitz et al., 2015). An important distinction characterizing the term in global health is the aspect of transfer of a practice from a low to high income setting which produces the same or better outcome for many at a notably lower cost (Cotton et al., 2014; Tran and Ravaud, 2016; Kulasabanathan et al., 2017). However, in 2021 a healthcare sector-specific definition does not yet exist (Sounderajah et al., 2021). Even the term ‘reverse’ is controversial (Cotton et al., 2014; Harris et al., 2016b; Bhattacharyya et al., 2017), as it implies that innovation normally flows unilaterally from high to low-income settings. Perpetuating this colonial perspective, in fact, undermines the shift in knowledge translation that the process should promote (Skopec et al., 2019; Harris et al., 2020).

While there is certainly ambiguity, lack of clarity, and lack of consensus regarding terminology, the idea that innovations in low-income countries are promising for adoption in high-income contexts is not new. Although reverse innovation was never mentioned, dozens of health innovations were curated in the early 1990s (Morgan and Rau, 1993) and BMJ published an entire issue one decade later profiling learning from low-income countries (Kumar, 2004). Nonetheless, in healthcare literature globally, 75% of publications that refer to ‘disruptive innovation’ were published since 2012 (Sounderajah et al., 2021). To assess the potential of innovative developments and technologies for improving animal health, we initiated a literature review in 2020. Contemporary intercultural and multi-language competence are central elements for global partnerships to leverage reverse innovation as global innovation (Zinsstag et al., 2019). The latter term contributes to “de-colonize” the flow of innovation and change prevailing mindsets (Crisp, 2015), promoting the urgently needed wide-ranging cooperation necessary to face crises with planetary implications like climate change and the current Covid-19 pandemic.

Such an urgently needed global cooperation clearly expands beyond animal health and includes human health and other related sectors as “One health”. We understand “One health” as the added value or incremental benefit of closer cooperation between human and animal health and related disciplines. Such added value can be generated in terms of better health of animals and humans, financial savings or environmental services that could not be achieved if the different sectors work alone (Zinsstag et al., 2021). Recent reviews already addressed issues with terrestrial implications like climate change and One health (Zinsstag et al., 2018) and the prevention of future pandemics (Zinsstag et al., 2020).

In this review, we aim to summarize the knowledge from innovations resulting from low-income, fragile contexts, where there is lack of trust, conflict and harsh climatic conditions with recurrent drought and flooding, which are all drivers for unconventional approaches and clean slate innovations, particularly for animal health but also from a One Health perspective.

## 2 Methods

PubMed, Web of Science and Scopus databases were considered as sources to identify peer reviewed published manuscripts related to reverse innovation and animal health. A series of test searches compared retrieved hits, duplicates and presence or absence of pre-identified relevant publications to refine the search strategy. Based on pragmatic consideration of feasible resources, we utilized a combined approach incorporating targeted searching with a snowball technique. The PubMed database was searched on October 20, 2020 using the developed key word algorithm. The final search string (Table 1) included Medical Subject Headings ("diffusion of innovation∗" + "developing countr∗"). The searched terms included: "reverse innovat∗" OR "animal monitor∗" OR (digital∗ + innovate∗) OR (sensor + "animal health") OR ("animal health" + ("rapid diagnostic test" OR "point of care test")) OR (POC + animal) OR (cross sector economic assessment + animal) OR ((("health plan∗") AND decentrali∗) + tool). The title/abstract filter was applied for the terms “animal” and “animal health”. An additional search was done using the term “contact sensor” with title/abstract filter on April 9, 2021. No date or language filters were applied.

**Table 1:** PubMed search strategy.

Filter	Term	Boolean operator
MESH	"diffusion of innovation∗"	AND
MESH	"developing countr∗"	OR
ALL	"reverse innovat∗"	OR
Title/Ab	"animal monitor∗"	OR
ALL	digital∗	OR
ALL	innovate∗	AND
ALL	sensor	OR
Title/Ab	"animal health"	AND
Title/Ab	"rapid diagnostic test"	OR
Title/Ab	"animal health"	AND
ALL	"point of care test"	OR
Title/Ab	"animal health"	AND
ALL	POC	OR
Title/Ab	animal	AND
ALL	cross sector economic assessment	OR
Title/Ab	animal	AND
ALL	“health plan”	OR
ALL	decentrali∗	AND
ALL	tool	AND

**Figure 1:**
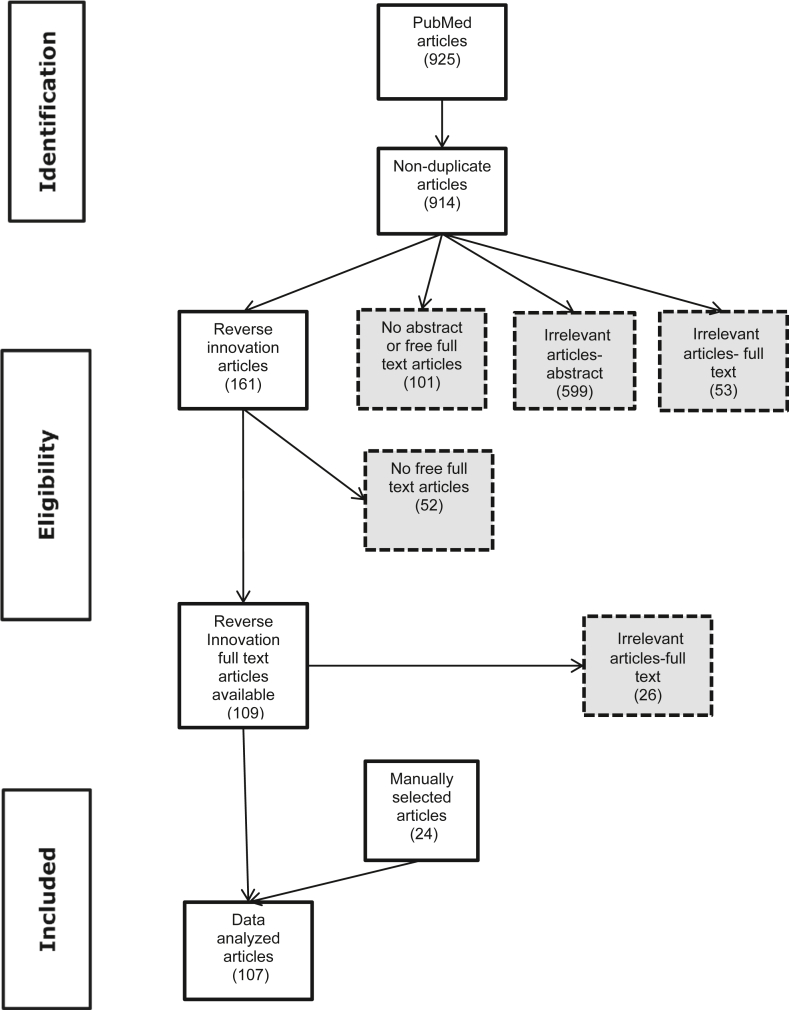
Flow diagram of review article screening.

EndNote™ bibliographic software was used to manage the citations. Duplicate articles were removed. Title and abstract were reviewed to identify irrelevant studies. Inclusion criteria were that the article mentioned or was related to reverse innovation or described conditions, processes or examples related to diffusion of innovation. Excluded were articles with both no abstract and no free full text available. Questionable articles were further included for full text review. During the full text review, cited relevant papers on reverse innovation were added using the snowball technique. Figure 1 shows the flow diagram for identifying and screening articles. Atlas.ti™ 7 software was used to extract data for qualitative coding from the selected articles.

## 3 Results

### 3.1 Literature search

The search strategy identified 925 articles, of which 11 were duplicates. Based on abstract review, 599 were categorized irrelevant based on title/abstract screening. Of articles with no abstract (171), available full texts were obtained (70). Of these, 53 were deemed irrelevant. Of the remaining 161 articles, 52 had no freely available full text and 26 were considered irrelevant. Another 24 articles were manually selected through snowballing, so in total, 107 articles were included for data analysis. One reviewer assessed all articles, while two additional reviewers assessed subsets of the identified articles. Disagreements were resolved through consensus. The heterogeneity of identified articles precluded meta-analysis, and quality scoring and risk of bias analysis were not feasible. Table 2 lists identifying details and topics for articles selected for data extraction.

**Table 2:** Identifying details of articles selected for data extraction.

First author	Year	Title	Study type	Innovation
**Targeted Search**				
Abadía-Barrero	2018	Kangaroo Mother Care in Colombia: A Subaltern Health Innovation against For-profit Biomedicine	Ethnographic	Healthcare provision
Arasaratnam	2013	Emerging economies drive frugal innovation	Commentary	Device
Arnold	2007	Knowledge translation in international emergency medical care	Review	Emergency medical care
Babo Martins	2016	Economic Assessment of Zoonoses Surveillance in a 'One Health' Context: A Conceptual Framework	Cost analysis	Framework for zoonotic disease surveillance
Babo Martins	2017	Economics of zoonoses surveillance in a 'One Health' context: an assessment of Campylobacter surveillance in Switzerland	Cost analysis	Zoonotic disease surveillance
Basu	2017	The role of South-North partnerships in promoting shared learning and knowledge transfer	Commentary	Global partnership
Bertrand	2004	Diffusion of innovations and HIV/AIDS	Review	HIV/AIDS prevention
Bhattacharyya	2017	Criteria to assess potential reverse innovations: opportunities for shared learning between high- and low-income countries	Modified Delphi	RI criteria
Binagwaho	2013	Shared learning in an interconnected world: innovations to advance global health equity	Commentary	Innovative partnership
Bloom	2018	Health system innovations: adapting to rapid change	Commentary	Health systems
Bolton	2019	Disseminating technology in global surgery	Review	Technology, medical device
Busse	2014	Learning from developing countries in strengthening health systems: an evaluation of personal and professional impact among global health volunteers at Addis Ababa University's Tikur Anbessa Specialized Hospital (Ethiopia)	Cross sectional survey	Global health partnership
Cordero	2008	Funding agencies in low- and middle-income countries: support for knowledge translation	Key Informant Interview	Knowledge translation
Correa	2004	Ownership of knowledge-the role of patents in pharmaceutical R&D	Commentary	Barriers to RI
Cotton	2014	Value innovation: an important aspect of global surgical care	Commentary	Surgical
Crisp	2014	Mutual learning and reverse innovation-where next?	Commentary	Mutual learning
Crisp	2015	Co-development, innovation and mutual learning-or how we need to turn the world upside down	Commentary	Mutual learning
Dalton	2017	Enablers of innovation in digital public health surveillance: lessons from Flutracking	Commentary	Opportunities for RI
De Paula Vieira	2020	Recalibrating Veterinary Medicine through Animal Welfare Science and Ethics for the 2020s	Commentary	Smart (precision) technology
DePasse	2013	A model for 'reverse innovation' in health care	Commentary	RI framework
Firoz	2017	Reverse innovation in maternal health	Case studies	Devices, mobile health, geographic information system
Fiz	2004	Non-invasive monitoring of diaphragmatic timing by means of surface contact sensors: an experimental study in dogs	Pilot study	Device
Foltz	1993	Modeling technology transfer in health information systems. Learning from the experience of Chad	Case report	Health information technology
Fraser	2005	Implementing electronic medical record systems in developing countries	Review	Electronic medical records
Free	1993	Health technologies for the developing world. Promoting self-reliance through improving local procurement and manufacturing capabilities	Review	Opportunities for RI
Fritz	2015	Success criteria for electronic medical record implementations in low-resource settings: a systematic review	Review	Electronic medical records
Harris	2000	Health technology transfer	Commentary	Technology transfer
Harris	2016	Does the Country of Origin Matter in Health Care Innovation Diffusion?	Commentary	Barriers to RI
Harris	2020	Review of the reverse innovation series in globalization and health - where are we and what else is needed?	Review	RI lessons and opportunities
Harris	2017	Measuring the bias against low-income country research: an Implicit Association Test	Cognitive psychology survey	Barriers to RI
Harris	2015	'They hear "Africa" and they think that there can't be any good services'-perceived context in cross-national learning: a qualitative study of the barriers to Reverse Innovation	Key informant interview	Barriers to RI
Harris	2016	That's not how the learning works - the paradox of Reverse Innovation: a qualitative study	Key informant interview	Barriers to and opportunities for RI
Ibe	2018	From Kisiizi to Baltimore: cultivating knowledge brokers to support global innovation for community engagement in healthcare	Randomized controlled trial	Community knowledge brokers
Johnston	2004	The cost-effectiveness of technology transfer using telemedicine	Cost analysis	Telemedicine, technology transfer
Kayali	2006	Cost-description of a pilot parenteral vaccination campaign against rabies in dogs in N'Djaména, Chad	Cost analysis	One health vaccination
Kulasabanathan	2017	Do International Health Partnerships contribute to reverse innovation? a mixed methods study of THET-supported partnerships in the UK	Mixed methods	International health partnership
Kumar	2016	I've got 99 problems but a phone ain't one: Electronic and mobile health in low and middle income countries	Commentary	e-health
Larios	2013	Five years of designing wireless sensor networks in the Doñana Biological Reserve (Spain): an applications approach	Pilot study	Technology, device
Lewis	2012	E-health in low- and middle-income countries: findings from the Center for Health Market Innovations	Review	e-health
Lubaba	2015	Movement Behaviour of Traditionally Managed Cattle in the Eastern Province of Zambia Captured Using Two-Dimensional Motion Sensors	Cross-sectional	Device
Mabey	2004	Diagnostics for the developing world	Review	Diagnostic tests
Mateen	2019	Multiple sclerosis in resource-limited settings: Research opportunities in an unequal world	Commentary	Opportunities for RI
McPherson	2014	Hearing assistive technologies in developing countries: background, achievements and challenges	Review	Device technology
Melon	2009	A survey of South-North health biotech collaboration	Cross sectional survey	Global partnership
Miranda	2010	Exporting 'failure': why research from rich countries may not benefit the developing world	Commentary	Barriers to RI
Morel	2005	Health innovation networks to help developing countries address neglected diseases	Commentary	Opportunities for RI
Mormina	2019	Science, Technology and Innovation as Social Goods for Development: Rethinking Research Capacity Building from Sen's Capabilities Approach	Analytic framework	Knowledge transfer
Murphy	2004	Diffusion of innovations: family planning in developing countries	Commentary	Family planning
Narrod	2012	A one health framework for estimating the economic costs of zoonotic diseases on society	Cost analysis	Framework for zoonotic disease economic assessment
Nosratabadi	2020	Food Supply Chain and Business Model Innovation	Review	Food supply chain business model
Novillo-Ortiz	2018	Digital health in the Americas: advances and challenges in connected health	Cross sectional survey	e-health
Okafor	2014	Evaluation of the interferon-γ assay on blood collected at exsanguination of cattle under field conditions for surveillance of bovine tuberculosis	Case-control	Point of care diagnostic test
Ovretveit	2011	Widespread focused improvement: lessons from international health for spreading specific improvements to health services in high-income countries	Review	Innovation scale up
Peña-Mohr	1987	Distributing and transferring medical technology. A view from Latin America and the Caribbean	Commentary	Medical technology
Piette	2015	Mobile Health Devices as Tools for Worldwide Cardiovascular Risk Reduction and Disease Management	Review	Mobile health technology
Piot	2016	Innovating healthcare delivery to address noncommunicable diseases in low-income settings: the example of hypertension	Conference proceedings	Healthcare delivery
Premji	2016	Call to Action for Nurses/Nursing	Review	Global partnership
Rezaie	2012	Emergence of biopharmaceutical innovators in China, India, Brazil, and South Africa as global competitors and collaborators	Qualitative analysis	Biopharmaceutical
Salicrup	2006	Challenges and opportunities for enhancing biotechnology and technology transfer in developing countries	Review	Technology transfer
Santosham	1997	Oral rehydration therapy for diarrhea: an example of reverse transfer of technology	Expert panel	Oral rehydration therapy
Serageldin	2002	The rice genome. World poverty and hunger-the challenge for science	Commentary	Opportunity for RI
Shalloo	2018	Review: Grass-based dairy systems, data and precision technologies	Review	Precision technology
Sharifi	2013	E-health implementation challenges in Iranian medical centers: a qualitative study in Iran	Expert survey	e-health
Silva	2011	Health technology diffusion in developing countries: a case study of CT scanners in Brazil	Key informant interview	Health technology policy
Simba	2004	Application of ICT in strengthening health information systems in developing countries in the wake of globalisation	Commentary	Information communication technology
Skopec	2019	Delivering cost effective healthcare through reverse innovation	Commentary	Opportunities/barriers for RI
Sleator	2007	Not so neglected diseases	Commentary	Knowledge transfer
Snowdon	2015	Reverse innovation: an opportunity for strengthening health systems	Review	Opportunities for RI
Sobolski	2005	Technology licensing: lessons from the US experience	Commentary	Technology transfer
Spicer	2018	'The development sector is a graveyard of pilot projects!' Six critical actions for externally funded implementers to foster scale-up of maternal and newborn health innovations in low and middle-income countries	Semi-structured interviews	Healthcare innovation scale-up
Subramanian	2011	Do we have the right models for scaling up health services to achieve the Millennium Development Goals?	Review	Scale up
Syed	2013	Reverse innovation in global health systems: towards global innovation flow	Commentary	Global partnership
Thakur	2018	Artificial-Intelligence-Based Prediction of Clinical Events among Hemodialysis Patients Using Non-Contact Sensor Data	Case control	Device
Tomasi	2004	Health information technology in primary health care in developing countries: a literature review	Review	Health information technology
Tran	2016	Frugal innovation in medicine for low resource settings	Commentary	Lean tools, opportunistic solutions, contextualized adaptations, bottom-up innovation
Tuijn	2011	Data and image transfer using mobile phones to strengthen microscopy-based diagnostic services in low and middle income country laboratories	Pilot study	Information communication technology, mobile digital data platform
Tura	2010	A low frequency electromagnetic sensor for indirect measurement of glucose concentration: in vitro experiments in different conductive solutions	Pilot study	Device
van Dam	2017	Open-source mobile digital platform for clinical trial data collection in low-resource settings	Pilot study	Mobile digital data platform
Varghese	2004	Categorizing the telehealth policy response of countries and their implications for complementarity of telehealth policy	Review	National telehealth policies
Watts	2011	Lost in translation	Commentary	Challenges to RI
Yapa	2018	Implementation science in resource-poor countries and communities	Review	Implementation science, opportunities for RI
Zhou	2020	Systematic review of early abortion services in low- and middle-income country primary care: potential for reverse innovation and application in the UK context	Review	Service delivery
Zinsstag	2007	Human benefits of animal interventions for zoonosis control	Cross-sector economic assessment	Zoonotic disease control

**Snowball Search**				
Awoonor-Williams	2013	Lessons learned from scaling up a community-based health program in the Upper East Region of northern Ghana	Case report	Community based health service
Bhatia	2020	Need for integrated surveillance at human-animal interface for rapid detection & response to emerging coronavirus infections using One Health approach	Commentary	Integrated surveillance system
Bordier	2018	Characteristics of One Health surveillance systems: a systematic literature review	Review	Integrated surveillance system
Dobbins	2009	A description of a knowledge broker role implemented as part of a randomized controlled trial evaluating three knowledge translation strategies	Randomized controlled trial	Knowledge translation
Hadengue	2018	Reverse innovation: A systematic literature review	Review	RI framework
Haefeli	2019	Investigation of the behaviour of cows using geolocated contact sensors	Cross-sectional survey	Device
Häsler	2014	A one health framework for the evaluation of rabies control programmes: a case study from Colombo City, Sri Lanka	Cost analysis	One Health intervention
Howitt	2012	Technologies for global health	Expert report	Health technology
Johnson	2018	The challenges of implementing an integrated One Health surveillance system in Australia	Key Informant Interviews	Integrated surveillance system
Kumar	2004	Learning from low-income countries	Commentary	Community based health service
Laager	2018	The importance of dog population contact network structures in rabies transmission	Cross-sectional survey	Device
Léchenne	2016	Validation of a Rapid Rabies Diagnostic Tool for Field Surveillance in Developing Countries	Validation	Diagnostic test
Paternoster	2017	Economics of One Health: Costs and benefits of integrated West Nile virus surveillance in Emilia-Romagna	Cost analysis	Integrated surveillance system
Roth	2003	Human health benefits from livestock vaccination for brucellosis: case study	Cost analysis	Integrated intervention
Schelling	2005	Synergy between public health and veterinary services to deliver human and animal health interventions in rural low income settings	Cost analysis	Integrated intervention
Schelling	2007	Towards Integrated and Adapted Health Services for Nomadic Pastoralists	Mixed methods	Integrated intervention
Sounderajah	2021	Are disruptive innovations recognised in the healthcare literature? A systematic review	Review	Categorized by domain
Stärk	2015	One Health surveillance - More than a buzz word?	Cost analysis	Integrated surveillance system
von Zedtwitz	2015	A typology of reverse innovation	Analytic framework	RI typologies
Wendt	2015	Zoonotic disease surveillance–inventory of systems integrating human and animal disease information	Review	Integrated surveillance system
WHO	2018	WHO compendium of innovative health technologies for low-resource settings	Compendium	Health technology
Zinsstag	2009	Transmission dynamics and economics of rabies control in dogs and humans in an African city	Cost analysis	Integrated intervention
Zinsstag	2019	Reverse innovation in global health	Commentary	Integrated approaches, global partnership
Zinsstag	2020	Towards integrated surveillance-response systems for the prevention of future pandemics	Commentary	Integrated surveillance systems

### 3.2 Analysis

The process of innovation diffusion has been discussed for decades. Earlier studies usually focused on characteristics of individual ‘trend setters’ in developing countries (Foltz, 1993; Bertrand, 2004). Later investigations broadened to understand how innovations and technologies were adopted, but the majority of these examined decision making in developed countries (Silva and Viana, 2011). DePasse and Lee (2013) advance a specific diffusion pathway for reverse innovation, postulating that because the learning is non-traditional, a ‘cross over’ is required. The social distance between LMIC early adopters and HIC innovators must be bridged by a ‘spannable distance’ (Kulasabanathan et al., 2017), necessitating involvement of diverse actors (policymakers, entrepreneurs, health system leaders) and channels (learning collaboratives, conferences, online resources). Such a concept of reverse innovation is much more fragmented and complex (Harris et al., 2016b). Investigators proposed a number of criteria to assess potential for reverse innovation (Snowdon et al., 2015; Bhattacharyya et al., 2017), shedding light on the process. Two central criteria are feasibility of the emerging market design and translation of innovation adoption. It becomes clear that innovation flow depends on co-development of ideas and mutual learning (Crisp, 2014). Integrated human and animal health studies among mobile pastoralists in Chad revealed that more cattle were vaccinated than children. Subsequent participatory transdisciplinary stakeholder meetings involving communities, authorities and scientists found consensus on the testing of joint human and animal vaccination services for mobile pastoralists in Chad (Schelling et al., 2007). These joint services saved up to 15% of financial resources compared to separate services and provided access to health care for previously inaccessible communities (Schelling et al., 2005).

#### 3.2.1 Influencing factors for reverse innovation

A first step promoting RI is awareness of potential opportunities within the health sector. Innovations can represent context-adapted alternatives with much improved cost-benefit ratios (Tran and Ravaud, 2016; Abadía-Barrero, 2018), increased quality of care for underserved areas (Sharifi et al., 2013), or optimized resource use (Snowdon et al., 2015; Premji and Hatfield, 2016; Basu et al., 2017; Zhou et al., 2020). Factors facilitating innovation can be less established infrastructure that allows more freedom to experiment (Crisp, 2014) and less retrofitting (van Dam et al., 2017) and an existing cultural ‘forgiveness of failure’ (Dalton, 2017). Global innovation flow requires bidirectional knowledge sharing (Crisp, 2014; von Zedtwitz et al., 2015) through equitable interdisciplinary partnerships (Syed et al., 2013; Basu et al., 2017). The roles of government are unique and cannot be overlooked (Dalton, 2017). Legislation and innovative policies impact diffusion of technology (Silva and Viana, 2011) and current funding allocations (Correa, 2004). Some argue that policies regulating social actions of governments must be underpinned by both economic and ethical obligation to health and well-being of citizens (Novillo-Ortiz et al., 2018; Sounderajah et al., 2021).

A number of innovation gaps are identified as motivators for RI. Price is a powerful driver, where ‘value for many’ requires a satisfactory performance at ultra-low cost (DePasse and Lee, 2013). Underdeveloped infrastructure provides a ‘clean slate’, where rapid implementation of new ideas is not hindered by existing behaviours (Morel et al., 2005; DePasse and Lee, 2013; van Dam et al., 2017). A third gap is less (or at least more protective/favourable) regulation (Correa, 2004; Salicrup and Fedorková, 2006; DePasse and Lee, 2013; Dalton, 2017). Additionally, both overwhelming needs and necessity for sustainability in low resource settings create a powerful incentive for innovation (DePasse and Lee, 2013). Funding also impacts innovation, with the last decades evidencing a shift from government supported research and development towards public institutions and the private sphere (Correa, 2004; Lewis et al., 2012), including, more recently, development agencies (Skopec et al., 2019). A knock on effect relates to competition, where publicly funded systems may be less incentivized to innovate than privatized ones (Snowdon et al., 2015).

Reverse innovation contributes both economic and societal advantages. Cost savings can be user-need driven to benefit individuals (Shalloo et al., 2018) or confer sector-wide and even cross-sectoral advantages, such as public and animal health initiatives for infectious disease control (Zinsstag et al., 2007; Babo Martins et al., 2017). The examples of rabies control (Roth et al., 2003; Kayali et al., 2006; Zinsstag et al., 2007; Häsler et al., 2014) and brucellosis (Roth et al., 2003; Zinsstag et al., 2007) demonstrate that animal interventions, while perhaps not being cost-effective from the animal or even the public health sector perspective, can save costs when all societal benefits are counted. Some RIs are somewhat less effective, but through significant cost reduction, reach more of a population and achieve a greater impact (Santosham et al., 1997; Cotton et al., 2014; Skopec et al., 2019). Reverse innovation additionally extends societal benefits from increased data security, accuracy and efficiencies like expedited validation (van Dam et al., 2017).

Along with opportunities for and advantages of reverse innovation, a number of challenges are elaborated. These primarily center on lacking information (Lewis et al., 2012; Sharifi et al., 2013; Sounderajah et al., 2021) and mistaken beliefs (Harris et al., 2015; Tran and Ravaud, 2016). Cultural considerations appear to play an important role. In terms of preferences, where acceptance impacts uptake both by decision makers and in communities (Morel et al., 2005; Spicer et al., 2018) and because the source of the innovation influences perception (Harris et al., 2016a). Implicit association stemming from cultural arrogance is noted to be a factor hindering uptake in HICs (Harris et al., 2017; Ibe et al., 2018). Change-resistant culture is also frequently mentioned as an obstacle (Cotton et al., 2014; Snowdon et al., 2015; Tran and Ravaud, 2016; Harris et al., 2020). Another hindrance to the process of reverse innovation can be systemic factors, such as infrastructural (Dalton, 2017) or bureaucratic inflexibilities (Snowdon et al., 2015), for example, in patenting processes (Correa, 2004).

#### 3.2.2 Characteristics of reverse innovation

The potential for reverse innovation is logically considered within the context of its characteristics. These are documented in the literature primarily as learning and knowledge. Potential exists when learning is bi-directional (Busse et al., 2014; Crisp, 2014), blank slate (DePasse and Lee, 2013), and collaborative or ‘jointly undertaken’ (Melon et al., 2009). Reverse innovation is facilitated when a mutual learning agenda is established based on need and opportunity (Kulasabanathan et al., 2017) in equity (Premji and Hatfield, 2016; Skopec et al., 2019). Multi-stakeholder participatory approaches involving communities generate essential learning through local adaptation and planning (Ovretveit, 2011), allowing crucial feedback (Bolton et al., 2019), and appear critical for innovation uptake and sustainability (Subramanian et al., 2011). However, collaboration is difficult in the face of complex systems and the associated large stakeholder groups (Snowdon et al., 2015), and, in particular, established power imbalances inhibit uptake of innovation (Kulasabanathan et al., 2017; Skopec et al., 2019). Scientific knowledge is a social good to be developed within a nation's innovation system, but alone it remains insufficient without the addition of informal knowledge, based on experiences, intuitions and interactions, which allow for transformational processes and products (Mormina, 2019). Innovation requires knowledge synthesis, exchange and application, which have together been construed as knowledge translation (Arnold et al., 2007). More recently, it is recognized that circulation and sharing of knowledge is non-linear, and knowledge translation is inherently complex two-way learning (Harris et al., 2016b). Knowledge brokers serve as intermediaries-individuals, organizations and structures-that network between innovator/developers, who create knowledge, and end users, who receive and translate knowledge into policies and practices (Dobbins et al., 2009; Ibe et al., 2018). von Zedtwitz et al. (2015) propose a map of global innovation, defining reverse innovations as those flowing from developing to developed countries at any phase of concept ideation, product development or introduction to primary/secondary markets.

Another important aspect is how to evaluate success of reverse innovation, but little is published on this. One study reviewed measurable criteria for successful implementation of electronic medical records (Fritz et al., 2015). Subsequently, a tool, including criteria for impact, cost, access, technical feasibility, and alignment with public policy, was proposed to screen innovations with high potential (Bhattacharyya et al., 2017). The term ‘scale-up’ is used in international health but, again, without an agreed definition (Ovretveit, 2011). Despite that, some case studies describe critical actions: identifying an exemplar, documenting essential features, communicating understandably, determining key factors for sustainability (Ovretveit, 2011). Innovation implementers should design for scale, generate evidence, use the power of individuals, and be responsive to ensure continuity (Spicer et al., 2018).

#### 3.2.3 Types of reverse innovation

A useful review of disruptive innovations (Sounderajah et al., 2021), published after our data collection timeframe but during data analysis, was identified through snowball technique. It categorized healthcare specific innovations into seven domains: basic science, devices, diagnostics, digital health, education, processes and techniques. Among the most cited innovations were ‘omics’ technologies, mobile health applications, telemedicine, and health informatics. The main conclusion was that ambiguous terminology leads to insufficient identification and thus poor understanding of the characteristics and, therefore, the potential of innovations. Other investigators also note these challenges, yielding a low number of documented examples to date (Crisp, 2014; Bhattacharyya et al., 2017; Skopec et al., 2019). Currently, several partnerships operate working groups which established online platforms to collate potentially adoptable innovations (Harris et al., 2016a, 2016b), for instance, the Centre for Health Market Innovations (CHMI) database (http://healthmarketinnovations.org/).

Our targeted review identified publications describing specific examples of reverse innovation from the above domains described by Sounderajah et al. (2021). Several device innovations were identified, including health monitors and sensors. Portable, durable low cost devices for cardiac monitoring during routine (DePasse and Lee, 2013) and maternal healthcare (Firoz et al., 2017) were developed for use in LMIC settings and subsequently spread to HICs. Non-contact sensors show potential for monitoring animal/patient vital parameters (Fiz et al., 2004; Thakur et al., 2018) and metabolites (Tura et al., 2010). Motion sensors (Lubaba et al., 2015) and wireless sensor networks (Larios et al., 2013) have been used advantageously to monitor and assess animal behaviour. Our research group collaboratively developed and pilot tested contact sensors on free roaming dogs in Chad to contribute knowledge on infectious disease transmission (Laager et al., 2018). These sensors were subsequently used to investigate behaviour of Swiss cows on alpine pastures (Haefeli, 2019). Regarding diagnostics, point of care tests were noted as having potential for reverse innovation but sparse evaluation in LMIC contexts (Mabey et al., 2004). Although our targeted search only identified one publication in this domain (Okafor et al., 2014), our research group contributed to validate a rapid rabies diagnostic test in Chad (Léchenne et al., 2016).

Processes included health care system innovations, and while there is a growing investment in and body of literature on these, outcomes, in terms of system change or improved health, are reported as disappointing so far (Bloom et al., 2018). They suggest that complexity necessitates special ‘systems approaches to health’ for diffusion at scale. Specific successes are noted for innovative programs to increase access and service delivery, for instance to address geographic barriers (Snowdon et al., 2015), or ‘task shift’ by training mid-level providers in maternal healthcare (Zhou et al., 2020) and community health workers in preventive care (Piot et al., 2016; Skopec et al., 2019). Other examples of process improvements are surgical innovations, many of which substitute a cheaper or easier, but equally effective, technique, tool or product (Cotton et al., 2014; Skopec et al., 2019). Decentralizing health care is an innovative process applied in Ghana, which progressed at different speeds across districts and with adaptations for local contexts, but is considered a successfully scaled initiative (Ovretveit, 2011; Awoonor-Williams et al., 2013).

Integrated surveillance systems are another process innovation which can enhance health outcomes through faster detection and cost savings (Narrod et al., 2012). Such systems employ cross- or multi-sectoral information sharing, which varies depending on the purpose, structure and sources of information (Babo Martins et al., 2017). About 60% of infectious organisms affecting humans are zoonotic, and 75% of human emergent diseases originate from animal reservoirs (Taylor et al., 2001). Annually, 2.5 million people worldwide die from zoonotic diseases (Grace et al., 2012). The global burden for rabies alone accounts is 1.6 million disability-adjusted life years (Coleman et al., 2004). In addition to public health consequences, emerging zoonotic diseases cause significant and widespread adverse economic impacts. A holistic one health approach where human health, animal health, the environmental sector, and related other disciplines collaborate for integrated disease surveillance and response is an effective way to address emerging and re-emerging zoonotic pandemics (Bhatia, 2020; Zinsstag et al., 2020). Since 2010, the tripartite organizations, the WHO, FAO and OIE, have encouraged multi-sectoral collaboration to reduce the public health and economic impact of diseases arising at the human-animal-environment interface (WHO, 2017). Although one health approaches have been recommended by international organizations and scientists, most existing surveillance systems still operate separately with no cross-sectoral collaboration for early detection of zoonotic disease (Wendt et al., 2015). The recent global pandemic of COVID 19 illustrates a clear failure of early detection of disease at the human-animal-environment interface due to an insufficient surveillance system.

Integrated surveillance systems have been implemented in several settings to halt the spread of zoonotic disease to the human population, including West Nile virus, avian influenza, Rift Valley fever and rabies (Bordier et al., 2018). Though there is evidence that early detection of disease in animals reduces the human burden of disease associated with zoonotic pathogens, which paramount from a public health point of view, a system's cost effectiveness is crucial to convince policy makers at the national and international levels and provide for sustainability. Cross-sector economic analyses can demonstrate that an earlier intervention, while the disease remains primarily in the animal reservoir, is less costly than interventions focused solely on human health (Zinsstag et al., 2009). Additionally, if zoonotic disease is detected earlier in animals or the environment, the cumulative cost remains lower than when disease spreads widely to humans (Zinsstag et al., 2020). One example, in the Emilia region of Italy, used information collected from animal health and environmental surveillance to guide a public health intervention, reducing the risk West Nile virus transmission through donated blood. Most of the averted cost arose from blood screening tests, cost of hospitalization and blood donation compensation (Paternoster et al., 2017).

The main issues slowing implementation of integrated surveillance systems include technical obstacles, such as data storage and sharing mechanisms or ethical concerns; budget constraints; existing silos, for example, regarding funders or communication; and low levels of political commitment (Bordier et al., 2018; Johnson et al., 2018). These challenges must be bridged through participatory discussions using cross sectoral platforms at national, regional and international levels. Using these collaborative activities, solid cost-benefit effectiveness of integrated surveillance systems can be demonstrated to further convince policy makers at different decision-making scales (Stärk et al., 2015).

### 3.3 Case study 1: integrated surveillance-response system in Somali Regional State, Ethiopia

A small scale integrated-surveillance response system (iSRS) at community level has been implemented in the Adadle district of the Somali Regional State of Ethiopia. To strengthen the existing government surveillance system in an innovative manner, staff of the Ethiopian Somali Regional State Livestock, Crop and Rural Development Bureau began to share an office, and together they collect data on human and animal health disease outbreaks, illness reports and emergency calls. They share their reports each day and discuss the respective relevance for their sectors. Reports come in directly from community animal health or community health workers by mobile phone calls or text messages. The team then organizes sample collection or assists with emergency medical evacuations where needed. Samples are sent to the University of Jigjiga (SRS), the Ethiopian Public Health Institute or the National Animal Health Diagnostic and Investigation Centre in Addis Ababa. Responses are organized through regional government public and animal health authorities.

The majority of the specific examples identified in our targeted search are categorized in the domain of digital health, which is unsurprising given the explosion in technologies and mobile phone uptake across even LMICs in the last decade. Breakthroughs in information and communications technology (ICT), and smart phones in particular, provide attractive avenues to address problems in health care accessibility, quality, effectiveness, efficiency and cost (Tuijn et al., 2011; Lewis et al., 2012; Piette et al., 2015). E-health, defined as use of ICT for health (https://www.who.int/ehealth/about/en/), encompasses a range of opportunities for reverse innovation. Digital health platforms enable high quality data collection (van Dam et al., 2017) and aid decision making for both providers and consumers (Firoz et al., 2017), while implementation appears independent of income level (Novillo-Ortiz et al., 2018). Electronic medical records are one example which have been successfully developed over recent years for diverse needs in different low-resource contexts (Tomasi et al., 2004; Fraser et al., 2005; Fritz et al., 2015). Mobile networks have also been utilized for knowledge sharing or quality control, for example sending diagnostic images (Tuijn et al., 2011). Major challenges to implementation of ICT programs identified were heavy reliance on donor funding, sustainability and human resource constraints (Lewis et al., 2012; Kumar et al., 2016), which likely impact both scale up and potential for reverse innovation. Although this review did not specifically target smart technologies, recent publications were identified (Shalloo et al., 2018; De Paula Vieira and Anthony, 2020) which emphasized the need for further cost-benefit analysis.

### 3.4 Case study 2: block Rabies-cutting edge digital tools for the one health context in LMICs

Dog mediated rabies is the main cause of human rabies, responsible for nearly 60,000 human deaths per year globally (Hampson et al., 2015). Nearly all of those cases occur in LMICs, with children being most affected (WHO, 2006; Banyard and Fooks, 2011; Hampson et al., 2015). Highly effective human post exposure prophylaxis (PEP) allows this fatal disease to be 100% preventable. PEP consists of local wound care, a series of three vaccinations and immunoglobulin application. However, for affected persons in LMICs, PEP is often unavailable due to deficient vaccine supply chains and inadequate communication between public health and veterinary services leading into wastage of expensive and scarce vaccines. Another reason is poorly informed bite victims, which results in not visiting a health center after a bite from a suspected animal (Bourhy et al., 2010; Dodet et al., 2010, 2015). Most LMICs have poor animal rabies surveillance, with rabies cases often recorded on paper, and the real burden of rabies remains unknown.

As a case example of RI, we profile the BlockRabies project. BlockRabies is funded by the European and Developing Countries Clinical Trials Partnership and runs in Mali and Côte d’Ivoire from 2020-2024. The BlockRabies application is the core of a unique and highly innovative blockchain (BC) secured One Health intervention approach. The application combines the public health sector, the veterinary sector, the Health Information system (HIS) and the vaccine supply chain. In addition, it enables high-level communication between the veterinary and health authorities, while respecting the security and confidentiality of the data. At the health centre, every human bite victim receives an electronic health record (EHR). In parallel, the status of the biting animal is recorded by the veterinary authorities and transferred to the physician treating the bite victim. The BockRabies application also sends vaccination reminders to patients, an important advantage ensuring that the lifesaving booster vaccinations are not forgotten. Information on patient compliance and clinical outcomes is continuously monitored and analysed. Since the vaccine supply chain is also integrated into the application, vaccine bottlenecks are avoided altogether through continuous vaccine inventory and automatic reordering. Selected information from the application is BC secured. Why do we propose to secure certain information on a BC in the LMIC context? The system consists of an open network of independently operated internet nodes all running the same node software, which communicate with each other to relay state updates in the form of transactions and generate a series of timestamped ‘blocks’ of transactions. These characteristics of a BC-based distributed ledger (data storage) enable novel forms of transparent data processing, with the useful properties of being immutable, decentralized and resistant to tampering. Another advantage is the availability of real time data. This is particularly relevant for contexts where there is lack of trust and unreliable existing information systems. Furthermore, unlike in centralized systems, power outages do not shut down an entire system. Additionally, predefined stakeholders can easily access the information. But along with the technical feasibility, the accompanying social, legal and ethical aspects of this innovative cutting edge technology is assessed. Through transdisciplinary processes with engagement of relevant stakeholders from the academic and non-academic sectors, important social knowledge will be generated and evaluated for successful technology modification, implementation and sustainability. Questions arising around data protection and data privacy are crucial and must be addressed and managed. The BlockRabies Coordination Board currently develop a BC ethical design framework for the BlockRabies project, as introduced by Lapointe and Fishbane (2019).

The objective of BlockRabies is to produce comprehensive proof of concept for this highly innovative BC-secured One Health application with integration of the entire vaccine supply chain and HIS in Côte d’Ivoire and Mali. Its implementation should markedly strengthen weak rabies health care systems in Africa, resulting in higher vaccination coverage of rabies-exposed persons. The BlockRabies application has potential to be implemented globally and be extended to other zoonotic diseases. Electronic health records are currently in quite an early stage even in high-income settings such as in Switzerland, and implementation in our partner countries would provide a successful example. In-depth analysis should still be performed in each context and the application should be adapted accordingly for optimal use. Such RI examples from LMICs can lead the way for HICs, which are often slow to move away from existing systems and processes (Howitt et al., 2012). Legal and regulatory barriers often hinder successful implementation of EHRs in HICs. The efforts to increase vaccination coverage for PEP and experiences implementing dog rabies mass vaccination campaigns identify understandable information to vaccine users as a central element for high vaccination coverage. These observations resulted from systematic approaches to intervention effectiveness during dog rabies mass vaccination campaigns (Muthiani et al., 2015; Mosimann et al., 2017). The thereby developed concept of intervention equity-effectiveness (Zinsstag et al., 2011) is clearly applicable in HICs in the current Covid-19 pandemic vaccination hesitancy context.

## 4 Discussion

This review was initiated to assess the potential of innovative developments and technologies for improving animal health. In order to consider potential, the scope was widened to describe and position reverse innovation in context. We largely limited the examples to consider reverse innovations in health. This targeted review of published literature is a gap analysis. The broadly heterogeneous nature of the topic of reverse innovation is evidenced by the still ambiguous terminology and the highly different but equally relevant disciplines, including health services research, management science, diffusion of innovation theory, organizational behaviour, cognitive psychology, marketing science, development studies and more (Harris et al., 2020). Although the concept of innovation diffusion is not new, establishing a global shared vocabulary and standardization is slow and continues to evolve (Sounderajah et al., 2021). We know from our own body of published research that relevant examples of successful or potential innovations will have been overlooked because authors fail to state specific terms describing reverse innovation or diffusion of innovation. This limits availability of robust systematic reviews, or even evaluations, on the topic. Another limitation is publication bias. Journal editors face constraints and elect to present only work that is deemed broadly interesting, which is often subjectively defined, and investigators only report on positive successes (Arnold et al., 2007; Fritz et al., 2015; Kulasabanathan et al., 2017; Skopec et al., 2019).

We can distinguish technical innovations like new digital devices, diagnostic tests and procedures, and social innovations of intersectoral cooperation, like One Health entailing joint human and animal health services or surveillance-response systems and participatory approaches leading to a better societal acceptance of novel interventions.

The way forward to a truly global flow of innovation must surely be through a multi-faceted approach. At baseline, are publications, such as those identified in this review, along with traditional reports, like the *WHO Compendium of innovative health technologies for low-resource settings,* listing available and prototype products and technologies from 2016-2017 (WHO, 2018). The compendium was published annually from 2011 through 2016. Efforts at open platforms to share ideas for consideration in relevant contexts already exist in early iterations, for instance, a “Compendium of Good Ideas” (http://frugal-innovation-medicine.com/) (Tran and Ravaud, 2016) and the CHMI resources and database noted above. There are also hubs focused on specific types of innovation, such as the World Health Organization's curating medical devices (https://www.who.int/health-topics/medical-devices). Challenges remain like oversight and evaluation, keeping entries up to date and sustainability. However, these are valuable initiatives towards establishing the formal forums that create mutually beneficial areas for idea exchange. A next step is continued development and use of criteria to screen for low-cost, high yield innovations (Bhattacharyya et al., 2017). We must develop and test methods to assess the effectiveness, impact and cost-effectiveness of implementing innovations (Aifah et al., 2019). Finally, only bi-directional partnerships and co-produced learning allow for identification of high-priority shared problems and create opportunities for change through global innovation (Crisp, 2015).

## 5 Conclusion

Innovation follows no borders and can also occur in low-income settings, specifically under constraints of cost, lack of services and infrastructure. Lower administrative and legal barriers may also contribute to innovations that would not be possible under conditions of high density of regulation. We recommend broadly adopting the term global innovation, which highlights those emanating from international partnership to solve problems with global implications.

## Declarations

### Author contribution statement

Lisa Crump, Yahya Maidane, Stephanie Mauti, Rea Tschopp, Seid Mohammed Ali, Rahma Abtidon, Hervé Bourhy, Zakaria Keita, Seydou Doumbia, Abdallah Traore, Bassirou Bonfoh, Mathilde Tetchi, Issaka Tiembré, Vessaly Kallo, Vega Paithankar, Jakob Zinsstag: Conceived and designed the experiments; Performed the experiments; Analyzed ​and interpreted the data; Contributed reagents, materials, analysis tools or data; Wrote the paper.

### Funding statement

This work was supported by Swiss Federal Food Safety and Veterinary Office and 10.13039/501100010473Federal Office for Agriculture (project number: 1.18.14TG).

### Data availability statement

Data referenced in article.

### Declaration of interests statement

The authors declare no conflict of interest.

### Additional information

No additional information is available for this paper.
